# Vaccination in Chronic Obstructive Pulmonary Disease

**DOI:** 10.3390/vaccines13030218

**Published:** 2025-02-22

**Authors:** Wang-Chun Kwok, Jin-Ning Clarabel Wong, Aaron Cheung, Terence Chi-Chun Tam

**Affiliations:** Department of Medicine, The University of Hong Kong, 102 Pokfulam Road, Pokfulam, Hong Kong SAR, China; kwokwch@hku.hk (W.-C.K.); clarabel1995@gmail.com (J.-N.C.W.); aaron_b_1992@hotmail.com (A.C.)

**Keywords:** influenza vaccine, pneumococcal vaccine, RSV vaccine, COVID-19 vaccine, zoster vaccine, pertussis vaccine, COPD

## Abstract

Chronic obstructive pulmonary disease (COPD) is often exacerbated by various viruses and bacteria, leading to acute episodes of worsening respiratory symptoms, which contribute significantly to the morbidity and mortality associated with COPD. Consequently, vaccination against these pathogens is recommended by numerous guidelines to safeguard COPD patients from adverse health outcomes. The Global Initiative for Chronic Obstructive Lung Disease (GOLD) recommendation advocates for vaccination against influenza, *Streptococcus pneumoniae*, respiratory syncytial virus (RSV), severe acute respiratory syndrome coronavirus (SARS-CoV2), pertussis, and varicella zoster. This review article will examine the current vaccination strategies recommended for adult COPD patients and will discuss the clinical benefits associated with these vaccines.

## 1. Introduction

Respiratory tract infections are common with chronic obstructive pulmonary disease (COPD) and were estimated to be the culprit in causing acute exacerbation (AECOPD) in up to 80% of cases [1]. Viruses can be detected in approximately two-thirds of AECOPD [2] while bacterial infections can be the identifiable trigger in up to half of these episodes [3,4,5,6]. COPD patients face an increased risk of severe respiratory infections caused by these pathogens; therefore, vaccination is crucial to prevent various adverse outcomes. In this review article, we will first examine the latest vaccination recommendation in adult COPD patients, followed by the evidence supporting each vaccine [7,8,9].

## 2. Global Initiative for Chronic Obstructive Lung Disease (GOLD)

The GOLD guidelines recommend vaccinations against influenza, severe acute respiratory syndrome coronavirus 2 (SARS-CoV-2), *Streptococcus pneumoniae*, respiratory syncytial virus (RSV), pertussis, and varicella zoster (VZV). The evidence levels for these recommendations are categorized as follows: Category A, which includes randomized controlled trials (RCTs) with high-quality evidence and minimal bias, applies specifically to the RSV vaccine, while Category B encompasses RCTs with significant limitations for the other vaccines [10]. A summary of the GOLD recommendation is provided in Table 1. The common and important adverse effects from these vaccines and their prevalence is summarized in Table 2.

## 3. Influenza Vaccine

Influenza is one of the most prevalent infectious respiratory viruses that causes significant morbidity, mortality, and healthcare burden. A preliminary estimation by the Centers for Disease Control and Prevention (CDC) on the in-season flu-related disease burden revealed up to 9.3 million flu illnesses, 140,000 flu hospitalizations and 13,000 flu deaths just from October–December 2024 in the US alone [11].

Studies have shown that seasonal influenza infection was a significant trigger for severe AECOPD, leading to increased rates of hospitalization, intubation, and mortality [12,13]. Influenza infection leads to increased airway inflammation, bacterial colonization, and immune dysregulation in COPD patients, resulting in severe exacerbations and poor outcomes [1]. Research indicates that COPD-related inflammation extends beyond the lungs, resulting in systematic effects as evidenced by an elevation of inflammatory markers such as C-reactive protein (CRP), interleukin (IL)-6, and tumor necrosis factor (TNF)-alphas [14]. As a result, COPD patients often have multiple medical comorbidities, with studies showing that up to 12% of those hospitalized for AECOPD experience an acute cardiac event [15,16]. The association between influenza infections and subsequent exacerbations underscores the critical need for preventive measures to reduce AECOPD, mortality and healthcare expenditure. The GOLD and American Thoracic Society (ATS) [17,18] guidelines strongly recommend annual influenza vaccine for all COPD patients regardless of COPD stages. The European Respiratory Society (ERS) guidelines [19] opined that influenza vaccination is a cost-effective way to reduce healthcare utilization.

Influenza viruses are classified into four types: A, B, C, and D, with type A being the most common pathogen. Influenza viruses are further subclassified based on the two surface antigens: Haemagglutinin (H) and Neuraminidase (N). The World Health Organization (WHO) recommends a specific strain of influenza for vaccine formulation in preparation for the upcoming flu season. For the 2025 influenza season in the northern hemisphere, the WHO has recommended the following:

- Egg-based Vaccines
  - A/Victoria/4897/2022 (H1N1)pdm09-like virus
  - A/Thailand/8/2022 (H3N2)-like virus
  - B/Austria/1359417/2021 (B/Victoria lineage)-like virus

- Cell Culture- or Recombinant-based Vaccines
  - A/Wisconsin/67/2022 (H1N1)pdm09-like virus
  - A/Massachusetts/18/2022 (H3N2)-like virus
  - B/Austria/1359417/2021 (B/Victoria lineage)-like virus

The Influenza vaccine is available in three main forms [20,21]: (1) Injectable Inactivated Vaccines (IIV), (2) Recombinant Vaccines (RIV), and (3) Live Attenuated Influenza Vaccine (LAIV). Injectable Inactivated Vaccines (IIV) are primarily egg-based, and they are suitable for most populations including those with compromised immune systems. Recombinant Vaccines (RIV) are created using recombinant DNA technology and cultured in mammalian cells, which helps avoid issues related to egg adaptation (i.e., mutations that can lead to discrepancies between the vaccine strain and circulating strains). Live Attenuated Influenza Vaccine (LAIV) is traditionally egg-based although some modern formulations may utilize cell culture methods; this is administered as a nasal spray and is not recommended for patients with compromised immune systems.

Older adults, immunocompromised, and COPD patients benefit from more potent vaccinations that offer enhanced immunogenicity. For instance, high dose inactivated influenza vaccines (HD-IIVs) contain four times the antigen of standard-dose inactivated influenza vaccines (SD-IIVs), and adjuvanted vaccines, such as those containing MF59 protein, enhance the immune response. These vaccines elicit a stronger immune response, which is critical for individuals with impaired immune function [22,23].

Barring some mild vaccine subtype-specific concerns, the vaccine is very safe [23,24]. Despite the involvement of egg products in the manufacturing of IIVs, IIV can still be safely administered in those with proven egg allergies. In general, contraindication to the injectables is with a history of severe allergic reaction to any component of the vaccine or to a previous dose of any influenza vaccine. For intranasal preparation, additional contraindication includes:

- Concomitant aspirin- or salicylate-containing therapy in children and adolescents
- Children aged 2–4 years who have received a diagnosis of asthma, or the child has a history of wheezing episodes during the preceding 12 months
- Children and adults who are immunocompromised due to any cause
- Close contacts of severely immunosuppressed persons
- Pregnancy
- Persons with active external CSF communication or any other cranial CSF leak
- Persons with cochlear implants
- Those who receive oseltamivir or zanamivir (within the previous 48 h), peramivir (within previous 5 days), and bloxavir (within the previous 17 days)

The types of influenza vaccines available are summarized in Table 3.

An early randomized, double-blind, placebo-controlled trial back in the 1990s already demonstrated the efficacy of the influenza vaccine in patients with COPD. The trial included 125 COPD patients who were randomized to receive either standard trivalent IIV or a placebo. The results showed that influenza vaccination effectively prevented influenza-related acute respiratory illness regardless of the COPD severity. Vaccine effectiveness was 84% for mild COPD [risk ratio (RR), 0.16 [*p* = 0.06]); 45% for moderate COPD [RR 0.55 (*p* = 0.5)] and up to 85% for severe COPD [RR 0.15 (*p* = 0.04)] [25]. Furthermore, a systematic review of six randomized controlled trials (RCTs) reinforced the importance of influenza vaccination to reduce AECOPD. The review consistently demonstrated a significant reduction in AECOPD for patients vaccinated with IIVs versus a placebo, with a weighted mean difference (WMD) of 0.37 [95% confidence interval (CI) −0.64 to −0.11, *p* = 0.006] [26].

A multi-centre RCT involving 31,989 patients in the United States and Canada compared the effects of standard trivalent and high-dose IIV. The findings revealed that older patients (≥65 years) experienced a significantly higher antibody response with the high-dose IIV. After two years, seroprotection against H1N1 was achieved in up to 98.5% in the high-dose group compared to 93.7% for the standard dose group, yielding a percentage point difference of 4.8 (95% CI = 4.1–5.5) [22].

Despite the proven benefits and satisfactory safety profile of the influenza vaccine, the vaccination rate remains low. A recent WHO report in 2023 shows that only 44.9% of adults in the United States (US) receive the flu vaccine annually [27]; this rate slightly rises to approximately 53.3% in US COPD patients [28]. Further public health efforts to increase accessibility with free vaccines, integration into routine COPD care, and digitalized reminders can help boost vaccination rates [29].

## 4. Pneumococcal Vaccine

Aside from viruses, bacterial infections are also prevalent in COPD patients. Pulmonary and systemic invasive infection due to *Streptococcus pneumoniae* are significant contributors to hospitalization and mortality in this population [30]. Invasive pneumococcal disease (IPD) continues to pose a serious health threat, with approximately 31,000 cases and 3590 deaths reported in the United States in 2017 alone [31]. Pneumococcal pneumonia occurs more frequently in COPD patients due to compromised mucociliary clearance and underlying structural lung damage [32]. Each episode of pneumonia sets off a cascade of negative health events. A retrospective cohort study found that the cumulative risk of COPD exacerbation in the first year following community acquired pneumonia (CAP) was significantly higher in patients who had had CAP (17% vs. 11.4%, *p* < 0.001) [33]. Each AECOPD leads to additional lung parenchymal damage and functional decline, making patients increasingly susceptible to further infections due to worsened mucociliary clearance and increased bacterial colonization [34]. This downward spiral hastens the disease course in COPD patients.

The are two main types of pneumococcal vaccines available for clinical use, namely pneumococcal polysaccharide vaccine (PPSV) and pneumococcal conjugate vассinе (РCV). The active components of both kinds of vaccine are capsular polysaccharides from pneumococcal serotypes that commonly cause invasive disease. PCV is the one currently recommended for COPD.

The cycle of pneumococcal pneumonia leading to AECOPD and subsequent COPD progression can be mitigated through effective vaccination strategies. The CAPITA trial, which involved over 84,000 participants in a randomized, double-blind, placebo-controlled study, demonstrated that the 13-valent Pneumococcal conjugate Vaccine (PCV13) effectively reduces the incidence of vaccine-type pneumococcal CAP (vaccine efficacy, 45.6%; 95.2% CI = 21.8–62.5; *p* < 0.001) [35]. Recent advancements have expanded the range of available pneumococcal conjugate vaccines, including PCV13, PCV15, and PCV20, each offering broader serotype coverage. The PCV vaccine elicit T-cell mediated responses that result in longer lasting immunity with memory effect. Conversely, the purified capsular polysaccharides-23 vaccine (PPSV23) provides a broader serotype coverage but induces a shorter-duration immunity due to T-cell independent B cell activation [36]. If PCV20 is administered, no booster is currently required; however, if PCV13 or 15 is given, then PPSV23 should be administered 1 year later (or at least 8 weeks later in immunocompromised patients). The GOLD guideline recognizes that the pneumococcal vaccine decreases the incidence of lower respiratory tract infection and recommends one dose of either PCV20 or PCV15 followed by PPSV23 for all COPD patients. The serotypes covered by different pneumococcal vaccine are summarized in Table 4.

A randomized controlled trial involving 596 COPD patients found that PPSV-23 demonstrated significant efficacy against CAP in patients less than 65 years of age (76%; 95% CI: 20–93%, *p* = 0.013). In patients with severe airflow obstruction, efficacy approached significance at 48% (95% CI: 27–80%, *p* = 0.076) as well [37]. Furthermore, systematic review encompassing 12 RCTs with up to 2171 COPD participants revealed that vaccination significantly reduced the likelihood of an AECOPD [odds ratio (OR) 0.60, 95% CI = 0.39–0.93; four studies, n = 446; GRADE: moderate) and the incidence of CAP (OR 0.62, 95% CI = 0.43–0.89; six studies, n = 1372; GRADE: moderate). However, vaccination did not significantly alter the incidence of specifically pneumococcal pneumonia (Peto OR 0.26, 95% CI = 0.05–1.31; three studies, n = 1158; GRADE: low) [38].

Despite the recognized link between pneumococcal pneumonia and AECOPD mortality, the vaccination rate among COPD patients remains low. A retrospective population-based cohort study in Budapest showed that only 10.8% of COPD patients received pneumococcal vaccines [39]. In Greece, only 32.5% of COPD patients aged 40–65 received any form of pneumococcal vaccine [40]. These figures may be misleadingly low as pediatric vaccination programs increasingly incorporate pneumococcal vaccines, potentially reducing adult vaccination rates. Nonetheless, public awareness regarding pneumococcus and its vaccine remains insufficient, as up to 54% of COPD patients in Hungary reported never having heard of pneumococcus [39]. To enhance pneumococcal vaccination rates among COPD patients, increased accessibility, multidisciplinary approach, and education initiatives are paramount. A pre-post intervention study in the US enrolled COPD patients into a post exacerbation outpatient care program, featuring rapid outpatient follow ups and nurse-led teleconsultations, demonstrated a significant increase in pneumococcal vaccination rates (37% vs. 82%, *p* < 0.001) [41]. Healthcare professionals must take the initiative to ensure that patients understand the critical role that vaccinations play in managing their condition and provide straightforward pathways for obtaining these essential immunizations.

## 5. RSV Vaccine

RSV is a significant pathogen responsible for a wide range of respiratory illnesses, from mild acute upper respiratory tract illness (ARI) to severe lower respiratory tract diseases (LRTD) such as bronchiolitis and pneumonia. Although RSV is most associated with severe disease in infants and young children, it also poses a significant risk to older adults and individuals with pre-existing health conditions.

Globally, RSV imposes a substantial healthcare burden for both outpatient and inpatient care. A multi-center study found that RSV accounts for 8.7% of outpatient-managed AECOPD [42]. Furthermore, a meta-analysis estimated that RSV-associated acute respiratory infections (RSV-ARI) lead to approximately 336,000 hospital admissions and 14,000 in-hospital deaths annually globally [43]. The risk of severe RSV disease escalates in older adults due to immunosenescence, which reduces the efficacy of RSV-specific T-cell responses, resulting in more severe manifestations of the disease [43].

Management for RSV-related illnesses primarily involves supportive care. As of 2024, GOLD recommends that individuals with COPD receive the RSV vaccine starting at age 60 [10]. All newly developed RSV vaccines target the F glycoprotein that is involved in the fusion of the viral envelope with the host cell membrane, allowing viral entry and replication. The F glycoprotein undergoes conformational changes that enable this fusion process, making it a key target for vaccines and therapeutic interventions aimed at preventing both RSV subtypes (A and B).

A recent international, placebo-controlled trial involving 24,966 participants demonstrated that a single dose of adjuvanted RSV prefusion F protein-based vaccine achieved 82.6% efficacy (96.95% CI = 57.9–94.1) against RSV-LRTD, 94.1% efficacy (95% CI = 62.4–99.9) against severe RSV-LRTD, and 71.7% efficacy (95% CI = 56.2–82.3) against ARI. Another bivalent RSV prefusion F protein-based vaccine trial recruited 34,284 participants and showed a vaccine efficacy of 66.7% (96.66% CI = 28.8–85.8) for mild illness and 85.7% (96.66% CI = 32.0–98.7) for severe illness [44]. Finally, an mRNA-based vaccine approved by the Centers for Disease Control and Prevention (CDC) demonstrated an vaccine efficacy of 83.7% (95.88% CI = 66.1–92.2) against RSV-LRTD with at least two signs or symptoms, while showing 82.4% (96.36% CI = 34.8–95.3) against disease with at least three signs or symptoms [45].

Clinical trials have indicated that protein-based RSV vaccines are generally well-tolerated; however, some serious adverse events, including Guillain-Barré Syndrome (GBS), have been reported with an estimated rate of fewer than 10 cases per one million vaccinations [46]. In January 2025, the FDA approved safety labeling changes to include a warning about the increased risk of GBS following vaccination.

The recent advancements in RSV vaccines represent a transformative milestone in the prevention and management of respiratory illnesses, offering enhanced protection against this virus. As vaccination programs are rolled out globally, ongoing research and monitoring will be pivotal to optimize vaccine efficacy, addressing challenges like waning immunity, and ensuring equitable access to these life-saving vaccines.

## 6. SARS-CoV-2 Vaccine

The coronavirus disease 2019 (COVID-19) pandemic, caused by the SARS-CoV-2 virus, poses significant challenges to individuals and healthcare systems globally, particularly for patients with COPD. These patients often experience reduced lung function, frequent exacerbations, and increased susceptibility to respiratory infections. COVID-19 primarily targets the respiratory system and can lead to severe complications such as pneumonia, acute respiratory distress syndrome (ARDS), and multi-organ failure. For individuals with pre-existing lung conditions like COPD, the risks are significantly heightened, resulting in higher rates of severe illness, need for intensive care, and mortality in this population [47,48]. The GOLD guideline emphasizes that COVID-19 vaccines are highly effective in preventing SARS-CoV-2 infection, and individuals with COPD are strongly encouraged to adhere to national recommendations [10].

A study conducted in Hong Kong demonstrated the vaccines CoronaVac and BNT162b2 exhibited moderate to high effectiveness against COVID-19-related outcomes. Specifically, after a two-dose CoronaVac, effectiveness against mortality, hospitalization, and severe complications were 77% (95% CI = 74–80%), 18% (95% CI = 6–23%), and 29% (95% CI = 12–43%), respectively. For the two-dose regimen of BNT162b2, the corresponding figures were 92% (95% CI = 91–94%), 33% (95% CI = 30–37%), and 57% (95% CI = 45–66%), respectively. Subsequent administration of a third and fourth dose further improve COVID-related outcomes [49]. In 2024, the CDC reported that the updated COVID-19 vaccine (monovalent XBB.1.5) provided an additional vaccine efficacy of approximately 54% against symptomatic SARS-CoV-2 infection compared to those that did not receive the updated vaccine [50].

While COVID-19 vaccines have played a pivotal role during the pandemic, non-pharmaceutical measures implemented to limit the spread of the SARS-CoV-2 virus, such as mandatory stay-at-home orders, compulsory facemask usage, and social distancing, have led to a notable decrease in the incidence of respiratory viral infections beyond COVID-19 [51,52]. A systematic review and meta-analysis across nine countries observed an overall 50% reduction in hospital admissions due to AECOPD, likely attributed to the decline in respiratory viral infections that typically trigger exacerbations [53].

Vaccine hesitancy remains a significant barrier to achieving widespread immunity, even among high-risk groups like COPD patients. A retrospective study examining COVID-19 vaccine hesitancy among U.S. adults from 2021 to 2022 revealed an increase in hesitancy from 86.6% to 92.4% among those who had not received any COVID-19 vaccines. Moreover, belief in the overall social benefit of the COVID-19 vaccine declined from 47.5% to 25.1% during this period [54]. Misinformation, fear of side effects, and distrust in healthcare systems contributed to this hesitancy. Healthcare providers play a crucial role in addressing these concerns by providing accurate information about vaccine safety and benefits while tailoring recommendations to each patient’s unique circumstances.

Even though COVID-19 vaccines have demonstrated high efficacy in preventing severe cases, their direct impact on COPD patients on a global scale remains to be fully explored. Future research should prioritize the collection of comprehensive data on how COVID-19 vaccination influences the prevalence of AECOPD while promoting ongoing vaccination efforts.

## 7. Pertussis Vaccine

Pertussis, commonly known as whooping cough, is a highly contagious respiratory infection caused by Bordetella реrtussiѕ [55]. Patients with COPD face an increased risk of contracting pertussis [56,57,58], and this is associated with higher healthcare costs and longer hospital stays [59]. While it has been suggested that influenza vaccination may elicit a weaker immune response in COPD patients [60], research has demonstrated that COPD patients exhibit robust immune responses to the pertussis vaccine characterized by induction of T-follicular helper cell, plasmablasts, and specific antibodies [61]. The GOLD guidelines recommended that unvaccinated COPD patients receive the reduced-antigen-content diphtheria-tetanus-acellular pertussis (Tdap) vaccine [10], aligning with recommendation form the CDC [62].

Despite the recognized need for pertussis vaccination among COPD patients, uptake remains suboptimal. A study analyzing data from a large USA administrative health claims system between 2008 and 2014 found that Tdap vaccination rates among COPD patients were lower than those in the general population (29.09, 42.26, and 39.03 per 1000-person years for COPD patients aged ≥65, 45 to 64, and 18–44, respectively, vs. 30.92, 54.05, and 52.90 for per 1000-person years for general population aged ≥65, 45 to 64, and 18–44, respectively) [63]. The barrier to vaccination includes misconceptions about pertussis disease, lack of awareness regarding vaccination requirements and insufficient recommendations from healthcare providers [64]. Given that unvaccinated COPD patients are more than twice as likely to contract pertussis, it is critical to enhance public awareness about the disease and promote vaccination efforts to improve uptake rates [57,65]. The increasing number of reported pertussis cases in the Asia–Pacific region further underscores the urgency of vaccinating vulnerable populations like those with COPD [66].

## 8. VZV Vaccine

Herpes zoster, resulting from the reactivation of the varicella-zoster virus (VZV), is reported to be more prevalent among patients with chronic medical diseases such as COPD [67,68,69,70,71]. The incidence may have particularly heightened in COPD patients receiving inhaled corticosteroid [70]. In addition to causing immediate dermatological symptoms, post-herpetic neuralgia is also an important complication that can result in persistent symptoms and disability [72]. In the United States, COPD patients who develop herpes zoster were shown to consume higher healthcare utilization and cost burden; specifically, the adjusted incidence rate ratio for all-cause and COPD-related healthcare utilization were found to be 1.17 and 1.27, respectively [73]. Additionally, the mean total all-cause cost was approximately USD 313 higher per person per month for all-cause and USD 152 higher for COPD-related costs [73].

VZV vaccination effectively reduces the risks of developing herpes zoster and post-herpetic neuralgia [74]. The GOLD guideline recommends VZV vaccination among COPD patients aged ≥50, aligning with recommendations from the CDC [74]. A multi-institutional propensity score-matched retrospective cohort study suggested that the recombinant zoster vaccine significantly lowers the incidence of herpes zoster in COPD patients, with a hazard ratio of 0.62 (95% CI = 0.51–0.75) for overall herpes zoster, 0.61 (95% CI = 0.49–0.75) for non-severe cases, and 0.53 (95% CI = 0.38–0.73) for severe cases [75]. Another retrospective study suggested that live attenuated zoster vaccine among patients could reduce the risks of myocardial infarction (OR 0.74, 95% CI = 0.66–0.83) and stroke (OR 0.75, 95% CI = 0.68–0.83) among patients with chronic diseases including COPD [76].

Despite these compelling findings, the awareness of zoster vaccine among healthcare professionals remains low. A study conducted in the United States reported that awareness levels ranged from 59.0 to 95.2%, with pulmonologists being the least likely to strongly endorse vaccination compared to other healthcare providers [77]. Among pulmonologists, only 41.1% agreed that herpes zoster and its complications warranted strong recommendations for vaccination (vs. 63.3% in family physicians, 59.2% in nurse practitioners, and 55.1% in physician assistants, *p* < 0.001).

While two-dose recombinant vaccine and one-dose VZV vaccines are available with Advisory Committee on Immunization Practices (ACIP) recommending the former [78], 30% of the pulmonologists, nurse practitioners and still report to recommend either the one- or two-dose VZV vaccine. 

Additionally, many COPD patients exhibit reluctance towards vaccination despite having a good awareness of the disease. A descriptive cross-sectional survey in Korea revealed that while 94.0% of people with self-reported COPD were aware of the VZV vaccine, only 33.1% had received it in accordance with ACIP recommendations [78,79]. Alarmingly, 74.7% were unaware that COPD increased their risks of developing herpes zoster. Exposure to educational materials—such as a brief video about the VZV vaccine–significantly increased interest in vaccination from 32.0% to 73.5%.

## 9. Specific Considerations

### 9.1. When to Vaccinate After AECOPD?

According to CDC recommendation, patients with mild illness can still receive influenza vaccination [80]. But for patients with moderate to severe illness, such as AECOPD, or patients with acute febrile illness, it is generally recommended that vaccination should be deferred until symptom resolution [80,81].

### 9.2. Vaccination Among Patients with Chronic Airway Colonization

As in other chronic respiratory diseases such as bronchiectasis, chronic airway infection and colonization with bacteria is also present in COPD, especially *Pseudomonas aeruginosa* [82,83,84]. While there is lack of evidence on safety of vaccination among COPD patients who have chronic airway infection and colonization, it is recommended that bronchiectasis patients should receive vaccines according to National Immunization Program Schedules, which include pneumococcal and influenza vaccine recommendations for high-risk patients with chronic lung disorders, as in the Thoracic Society of Australia and New Zealand (TSANZ) position statement on chronic suppurative lung disease and bronchiectasis in children, adolescents, and adults in Australia and New Zealand [85]. As such, it shall be considered safe for COPD patients who have chronic airway infection and colonization to receive vaccination as in bronchiectasis.

## 10. Future Development

In recent years, advancement in technology has led to the investigation of newer vaccines targeting additional respiratory pathogens, including the parainfluenza virus [86] and human metapneumovirus [86,87,88]. These viruses play a significant role in triggering AECOPD [89,90]. If these vaccines can be demonstrated to be safe and effective, they may offer substantial clinical benefits among COPD patients by further preventing viral triggered AECOPD.

## 11. Conclusions

In this review article, we provide a comprehensive summary of guideline recommendations for vaccination in COPD. Additionally, we present an overview of the evidence supporting the use of the individual vaccine in this population.

## Figures and Tables

**Table 1:** Vaccine recommended by GOLD for COPD patients.

Vaccine	Dosing and Frequency	Specific Scenario or Indication	Level of Evidence
Influenza	Annually	-	B
Pneumococcal		-	B
PCV 20	Once		B
PCV 15 followed by PPSV 23	ΡSV23 administered 1 year after PCV15 (or ≥8 weeks after PCV15 in patients with an immunocompromising condition, cochlear implant, or CSF leak)		B
Severe acute respiratory syndrome coronavirus 2	Two 2024–2025 formula, with the second dose given 2–6 months after the first dose	Age ≥ 65, immunocompetent	B
	One dose 2024–2025 formula	Age 5–64, immunocompetent	B
	At least three mRNA vaccine doses	Immunocompromised	B
Respiratory syncytial virus	Once	Age > 60	A
Pertussis	Once	For patients who were not vaccinated in adolescence	B
Varicella zoster	Two doses 2–6 months apart for recombinant vaccine	Age > 50	B

PCV: Pneumococcal conjugated vaccine, PPSV: Pneumococcal polysaccharide vaccine; CSF: Cerebrospinal fluid; mRNA: messenger ribonucleic acid.

**Table 2:** Adverse effects of the vaccines.

Vaccine	Common/Important Adverse Effects	Prevalence
Influenza		
Inactivated vaccines	Local injection site reaction (Pain, redness, swelling)	4–24%
	Shoulder bursitis	<0.01%
	Guillain-Barré syndrome	<0.01%
	Allergic reaction	<0.01%
Live attenuated vаϲϲiոе	Rhinorrhea, nasal congestion, headache, and sore throat	2–75%
Pneumococcal		
	Injection site reactions (Pain, swelling, induration)	15–20%
	Systemic symptoms (Fever, chills, fatigue, headache, myalgias, arthralgias)	<5%
Severe acute respiratory syndrome coronavirus 2		
	Injection site reactions	16–80%
	Fatigue, headache, and myalgia	40–60%
	Fever, chills, and joint pain	20–40%
	Guillain-Barré syndrome	<0.01%
	Thrοmboѕis with thrombocytopenia	<0.01%
	Myocarditis	Up tp 0.01%
Respiratory syncytial virus		
	Injection site reactions	6–76%
	Fatigue and headache	13–40%
	Arthralgia and myalgia	8–36%
	Diaarhoea, nausea, and vomiting	2–12%
	Guillain-Barré syndrome	<0.01%
Pertussis		
	Nausea	9–13%
	Injection site reactions	11%
	Bone pain	Up to 30%
	Chills	8–15%
	Fatigue/Malaise	13–33%
	Headache	12%
	Fever	Up to 2%
Varicella zoster		
	Gastrointestinal adverse effects	13–24%
	Injection site reactions	10–88%
	Myalgia	35–57%
	Shivering	11–36%
	Fatigue	37–57%
	Headache	15–51%
	Fever	6–28%

**Table 3:** Types of influenza vaccines.

Formulations	Age Indication	Route of Administration	Egg-Based/Culture-Based	Dosage
Inactivated influenza vaccine, trivalent	≥6 months	IMI	Egg-based	Standard-dose
	18–64 years			
	≥3 years			
	≥6 months	IMI (PFS)		
	≥6 months	IMI (PFS)		
	≥6 months	IMI (PFS or MDV)		
Cell culture-based inactivated influenza vaccine	≥6 months	IMI (PFS or MDV)	Cell culture-based	Standard-dose
High-dose inactivated influenza vaccine, trivalent	≥65 years	IMI	Egg-based	High-dose
Adjuvanted inactivated influenza vaccine, trivalent (MF59 adjuvant)	≥65 years	IMI	Egg-based	Standard-dose
Recombinant influenza vaccine, trivalent (recombinant hemagglutinin vaccine)	≥18 years	IMI	N/A	Standard-dose
Live attenuated influenza vaccine, trivalent	2–49 years	Intranasal spray	Egg-based	Standard-dose

IMI: intramuscular injection; MDV: multidose vial; NAS: intranasal; PFS: prefilled syringe.

**Table 4:** Serotypes covered by pneumococcal vaccines.

	1	2	3	4	5	6A	6B	7F	8	9N	9V	10A	11A	12F	14	15B	17F	18C	19A	19F	20	22F	23F	33F
PCV13	✔		✔	✔	✔	✔	✔	✔			✔				✔			✔	✔	✔			✔	
PCV15	✔		✔	✔	✔	✔	✔	✔			✔				✔			✔	✔	✔		✔	✔	✔
PCV20	✔		✔	✔	✔	✔	✔	✔	✔		✔	✔	✔	✔	✔	✔		✔	✔	✔		✔	✔	✔
PPSV23	✔	✔	✔	✔	✔		✔	✔	✔	✔	✔	✔	✔	✔	✔	✔	✔	✔	✔	✔	✔	✔	✔	✔

## Data Availability

This review article does not include data not presented in this article.
